# *Sod1* Loss Induces Intrinsic Superoxide Accumulation Leading to p53-Mediated Growth Arrest and Apoptosis

**DOI:** 10.3390/ijms140610998

**Published:** 2013-05-24

**Authors:** Kenji Watanabe, Shuichi Shibuya, Hirofumi Koyama, Yusuke Ozawa, Toshihiko Toda, Koutaro Yokote, Takahiko Shimizu

**Affiliations:** 1 Department of Advanced Aging Medicine, Chiba University Graduate School of Medicine, 1-8-1 Inohana, Chuo-ku, Chiba 260-8670, Japan; E-Mails: kng.wtnb@chiba-u.jp (K.W.); white10parakeets@hotmail.co.jp (S.S.); hkoyama@goo.jp (H.K.); ozawayusuke3@gmail.com (Y.O.); hik_toda@proteome.jp (T.T.); 2 Department of Clinical Cell Biology and Medicine, Chiba University Graduate School of Medicine, 1-8-1 Inohana, Chuo-ku, Chiba 260-8670, Japan; E-Mail: kyokote@faculty.chiba-u.jp

**Keywords:** reactive oxygen species, superoxide dismutase, vitamin C, p53, apoptosis

## Abstract

Oxidative damages induced by a redox imbalance cause age-related changes in cells and tissues. Superoxide dismutase (SOD) enzymes play a major role in the antioxidant system and they also catalyze superoxide radicals (O_2_^•−^). Since the loss of cytoplasmic SOD (SOD1) resulted in aging-like phenotypes in several types of mouse tissue, SOD1 is essential for the maintenance of tissue homeostasis. To clarify the cellular function of SOD1, we investigated the cellular phenotypes of *Sod1*-deficient fibroblasts. We demonstrated that *Sod1* deficiency impaired proliferation and induced apoptosis associated with O_2_^•−^ accumulation in the cytoplasm and mitochondria in fibroblasts. *Sod1* loss also decreased the mitochondrial membrane potential and led to DNA damage-mediated p53 activation. Antioxidant treatments effectively improved the cellular phenotypes through suppression of both intracellular O_2_^•−^ accumulation and p53 activation in *Sod1*-deficient fibroblasts. *In vivo* experiments revealed that transdermal treatment with a vitamin C derivative significantly reversed the skin thinning commonly associated with the upregulated p53 action in the skin. Our findings revealed that intrinsic O_2_^•−^ accumulation promoted p53-mediated growth arrest and apoptosis as well as mitochondrial disfunction in the fibroblasts.

## 1. Introduction

Reactive oxygen species (ROS) are mainly generated from mitochondrial respiration and they non-specifically oxidize cellular molecules including proteins, nucleic acid, and lipids, resulting in oxidative damage in organisms [1]. Redox balance is physiologically regulated through the production and degradation of ROS by antioxidant systems to protect cells from oxidative damage. Extrinsic excess ROS induces the DNA damage response (DDR) associated with oxidative DNA damage and promotes a canonical ATM-p53 cascade that regulates the cell fate [2]. ROS also dysregulates mitochondrial function through a reduction of membrane potential and respiration [3]. In this context, maintenance of redox balance in cells plays an important role in the determination of cellular fate and function, including apoptosis, cell cycle arrest, differentiation, and energy metabolism [4].

Superoxide dismutase (SOD) is one of the major antioxidant enzymes that catalyzes the conversion of superoxide radicals (O_2_^•−^) to hydrogen peroxide (H_2_O_2_) and O_2_[5]. SOD1 and SOD2 are ubiquitously expressed in tissues, and localized in the cytoplasm and mitochondria, respectively. Our previous studies demonstrated that *Sod1*-deficient (*Sod1^−/−^*) mice show various aging-like pathologies, such as: age-related macular degeneration [6], fatty deposits in the liver [7], skin atrophy [8], osteoporosis [9], deterioration of Alzheimer’s disease (AD) [10], luteal degeneration [11], and lacrimal degeneration [12]. Furthermore, several groups have reported that *Sod1^−/−^* deficiency also induced: hepatocellular carcinoma [13], muscle atrophy [14], hemolytic anemia [15] in mice, and poor growth rate in cells [16]. These observations indicate that *Sod1^−/−^* mice have the potential to be a valuable animal model for investigating human age-related diseases.

In the present study, we investigated the cellular phenotypes of *Sod1^−/−^* fibroblasts to clarify the biological significance of *Sod1* and the pathophysiological role of intracellular O_2_^•−^. We also investigated the involvement of the DDR and p53 activation under an intrinsic O_2_^•−^ accumulation. Finally, we have discussed the anti-aging effect of an antioxidant administered both *in vitro* and *in vivo*.

## 2. Results and Discussion

### 2.1. Sod1 Deficiency Induced Apoptotic Cell Death with Increased Superoxide Accumulation in Fibroblasts

**Figure 1:**
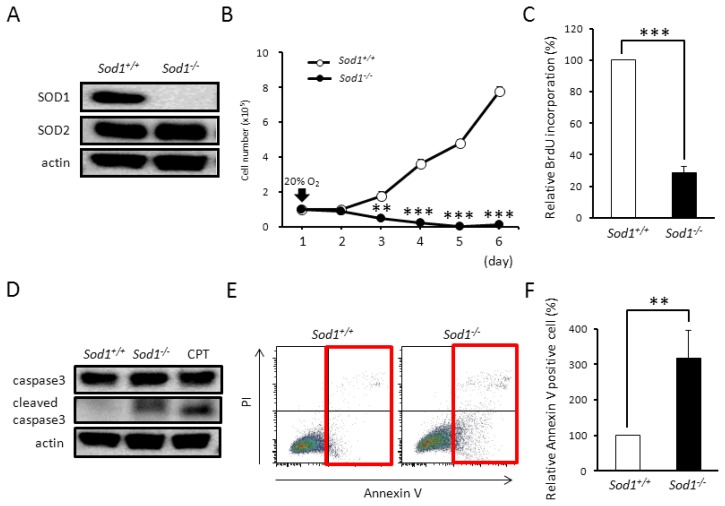
*Sod1* deficiency induces growth arrest and cell death in primary dermal fibroblasts. (**A**) SOD1 and SOD2 expression in *Sod1^−/−^* and *Sod1^+/+^* fibroblast. (**B**) The cell numbers of *Sod1^−/−^* and *Sod1^+/+^* fibroblasts (*n* = 3) were counted at the times indicated; (**C**) The cell viabilities of *Sod1^−/−^* and *Sod1^+/+^* fibroblasts (*n* = 3) were measured by BrdU incorporation; (**D**) Apoptosis in *Sod1^−/−^* and *Sod1^+/+^* fibroblasts was analyzed by western blotting with an anti-cleaved caspase3 antibody. To induce apoptosis, the cells were treated with 12 μM camptothecin (CPT) for 12 h; (**E**) Apoptosis in *Sod1^−/−^* and *Sod1^+/+^* fibroblasts was analyzed by flow cytometry with propidium iodide (PI) and Annexin V; (**F**) The relative percentage of Annexin V positive cells in *Sod1^−/−^* and *Sod1^+/+^* fibroblasts (*n* = 3). These data indicate the mean ± SD; ** *p* < 0.01, *** *p* < 0.001.

In order to investigate the biological significance of the SOD1 enzyme in cells, we analyzed the cellular phenotypes of *Sod1*-deficient primary dermal fibroblasts. Western blot analysis revealed the complete loss of the SOD1 protein in *Sod1^−/−^* cells (Figure 1A). Interestingly, the concentration of the SOD2 protein, an alternative intracellular SOD localized in mitochondria, remained unchanged in *Sod1^−/−^* cells, suggesting that SOD1 loss did not induce the compensatory expression of SOD2 protein in the cells (Figure 1A). Likewise, expression levels of other antioxidant enzymes, including glutathione peroxidase 1 and catalase, were not upregulated in *Sod1^−/−^* cells (data not shown). In cell culture experiments, *Sod1^−/−^* fibroblasts showed the marked loss of cell viability under a 20% O_2_ concentration (Figure 1B). We next analyzed the incorporation of BrdU to measure the proliferative ability of the *Sod1^−/−^* fibroblasts. As shown in Figure 1C, *Sod1* loss significantly impaired the incorporation of BrdU at culture day 2, indicating the disturbance of cell proliferation. Furthermore, *Sod1* depletion markedly increased the expression of cleaved caspase3 (Figure 1D) and annexin V positive cells (Figure 1E,F), indicating the induction of apoptotic cell death. These results demonstrated that *Sod1* deficiency induced proliferative decline and apoptosis in dermal fibroblasts.

**Figure 2:**
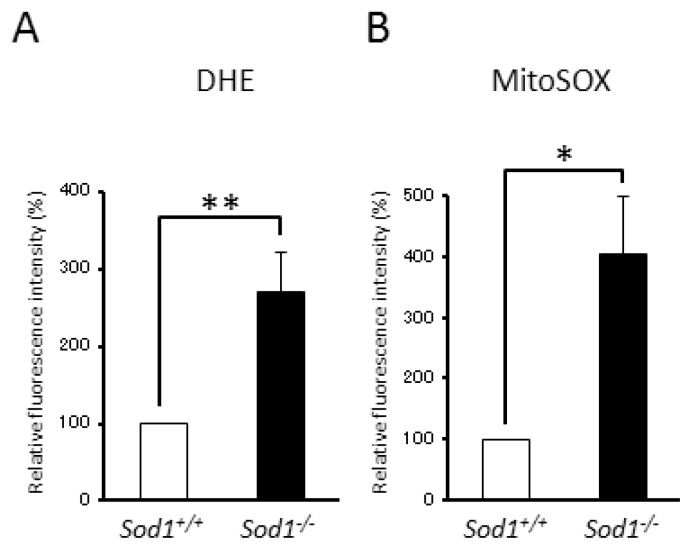
*Sod1* loss induces O_2_^•−^ generation and mitochondrial dysfunction in fibroblasts. (**A**,**B**) Intracellular O_2_^•−^ was measured by flow cytometry with dihydroethidium and MitoSOX in *Sod1^−/−^* and *Sod1^+/+^* fibroblasts (*n* = 3). These data indicate the mean ± SD; * *p* < 0.05, ** *p* < 0.01.

Because SOD1 catalyzes O_2_^•−^ to H_2_O_2_ and O_2_ in the cytoplasm, SOD1 loss results in increased cytoplasmic O_2_^•−^ accumulation in cells. In order to evaluate the O_2_^•−^ imbalance by SOD1 deficiency, we measured the O_2_^•−^ level using flow cytometry and a specific fluorescent dye for cytoplasmic O_2_^•−^, dihydroethidium (DHE). The DHE staining revealed a significant, 2.7-fold enhancement in the cytoplasmic O_2_^•−^ level in *Sod1^−/−^* compared to *Sod1^+/+^* fibroblasts (Figure 2A). Interestingly, MitoSOX staining, which is a specific fluorescent dye for O_2_^•−^ in mitochondria, also revealed a significant, 4-fold enhancement in the mitochondrial O_2_^•−^ level in *Sod1^−/−^* compared to *Sod1^+/+^* fibroblasts (Figure 2B), These results suggested that SOD1 regulates the O_2_^•−^ balance in both the cytoplasm and the mitochondria.

### 2.2. Sod1 Loss Caused p53 Upregulation Associated with Mitochondrial Dysfunction in Fibroblasts

**Figure 3:**
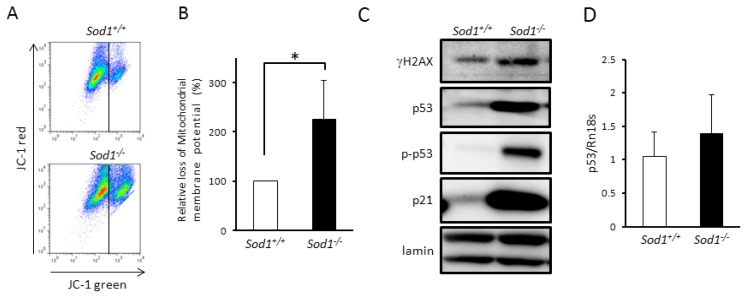
*Sod1* loss induces mitochondrial dysfunction in fibroblasts. (**A**) The loss of mitochondrial membrane potential (ΔΨm) was measured by flow cytometry with JC-1; (**B**) The relative percentage of mitochondria with low ΔΨm in *Sod1^−/−^* and *Sod1^+/+^* fibroblasts (*n* = 4); (**C**) Western blotting of DNA damage response proteins such as p53, p53 phosphorylation at Ser^18^, γH2AX and p21 in *Sod1^−/−^* and *Sod1^+/+^* fibroblasts; (**D**) p53 mRNA expression was analyzed by quantitative PCR in *Sod1^−/−^* and *Sod1^+/+^* fibroblasts (*n* = 4). These data indicate the mean ± SD; * *p* < 0.05.

Since mitochondrial ROS induces the loss of mitochondrial membrane potential (ΔΨm) [3], we measured ΔΨm using a JC-1 dye in *Sod1^−/−^* fibroblasts. As expected, *Sod1^−/−^* fibroblasts showed a 2.2-fold increase in the number of mitochondria with low ΔΨm (Figure 3A,B). Since decreased ΔΨm induces apoptosis [17], our findings suggested that O_2_^•−^ accumulation in mitochondria resulting from *Sod1* deficiency results in apoptosis through mitochondrial dysfunction.

Tumor suppressor p53 plays a crucial role in various cellular functions such as apoptosis, cell cycle arrest, energy metabolism, and senescence [4]. Since DNA damage caused by excess irradiation and ROS stimulates upregulation and phosphorylation of p53 via DDR resulting in apoptosis [18], we analyzed the p53 level in *Sod1^−/−^* fibroblasts. Western blot analysis clearly demonstrated markedly increased protein levels and phosphorylation at Ser^18^ of p53 in *Sod1^−/−^* fibroblasts (Figure 3C). Quantitative PCR analysis revealed that *Sod1^−/−^* deficiency had a tendency to increase p53 mRNA levels, but not significantly (Figure 3D), suggesting that p53 upregulation is not only regulated by mRNA levels but may be due to p53 stabilization in *Sod1^−/−^* fibroblasts. Furthermore, we found phosphorylated H2AX at Ser^139^ (γH2AX), a DNA damage marker, and identified the upregulation of p21, a target gene of p53 (Figure 3C). These data indicated that the DNA damage caused by *Sod1* deficiency also induced a proliferative defect and apoptosis via p53 activation.

### 2.3. A Vitamin C Derivative Rescued Viability of Sod1-Deficient Fibroblasts through a Suppression of O_2_^•−^ Generation and p53 Upregulation

**Figure 4:**
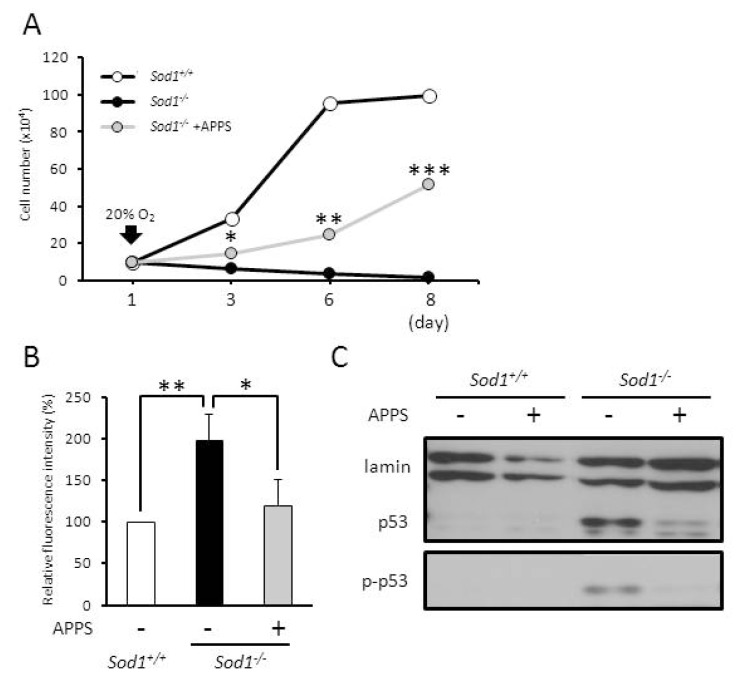

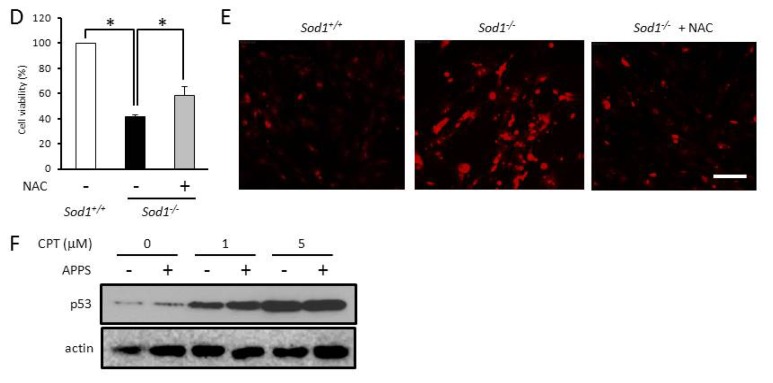
A VC derivative rescues cellular phenotypes in *Sod1^−/−^* fibroblasts. (**A**) The cell numbers of *Sod1^−/−^* fibroblasts treated with or without 10 μM APPS was counted at the time indicated (*n* = 3); (**B**) The relative O_2_^•−^ level in *Sod1^−/−^* fibroblasts treated with 10 μM APPS was measured by DHE (*n* = 3); (**C**) Western blot analysis of p53 and p53 phosphorylation at Ser^18^ in *Sod1^−/−^* fibroblasts treated with 10 μM APPS; (**D**) The cell viabilities of *Sod1^−/−^* and *Sod1^+/+^* fibroblasts (*n* = 3) were measured at culture day 2 by Cell Counting Kit-8; (**E**) Intracellular O_2_^•−^ level in *Sod1^−/−^* fibroblasts treated with 1 mM NAC was measured by DHE. The scale bar represents 100 μm; (**F**) Western blot analysis of p53 in NIH3T3 with or without 20 μM APPS. To induce DDR response, the cells were treated with CPT for 4 h. These data indicate the mean ± SD; * *p* < 0.05, ** *p* < 0.01, *** *p* < 0.001.

*In vitro* data indicate that the suppression of intracellular O_2_^•−^ generation and p53 activation by antioxidants can improve the cellular phenotypes of *Sod1^−/−^* fibroblasts. Thus, we evaluated whether antioxidants could protect the cellular phenotypes in *Sod1^−/−^* fibroblasts. To investigate the rescue activity of a vitamin C (VC) derivative, L-ascorbyl 2-phosphate 6-palmitate (APPS), we cultured *Sod1* null fibroblasts in the presence of APPS. The APPS treatment (10 μM) significantly increased the cell numbers of *Sod1^−/−^* fibroblasts at culture day 3 under a 20% O_2_ condition (Figure 4A). At culture days 6 and 8, *Sod1^−/−^* fibroblasts proliferated markedly in the presence of APPS compared to absence of APPS (Figure 4A). Finally, *Sod1^−/−^* fibroblasts with APPS propagated to confluence in a culture dish under a 20% O_2_ condition (data not shown), indicating that the VC derivative notably enhanced the viability of *Sod1^−/−^* fibroblasts. Next, we measured the intracellular O_2_^•−^ levels in *Sod1^−/−^* fibroblasts in the presence of APPS to confirm the anti-oxidative ability of APPS. Treatment with APPS completely suppressed the intracellular O_2_^•−^ level in *Sod1^−/−^* fibroblasts at culture day 2 (Figure 4B), indicating the effective anti-oxidant activity of APPS.

To investigate the downstream molecular events by *Sod1* deficiency, we assessed the p53 expression levels in APPS-treated *Sod1^−/−^* fibroblasts. Treatment with APPS significantly inhibited p53 upregulation and phosphorylation at Ser^18^ (Figure 4C). We also cultured *Sod1^−/−^* fibroblasts in the presence of another antioxidant, *N*-acetyl cysteine (NAC). As expected, treatment with NAC significantly accelerated cell viability and suppressed the intracellular O_2_^•−^ level in *Sod1^−/−^* fibroblasts (Figure 4D,E). Furthermore, to exclude the direct action of APPS on p53 expression, we investigated the inhibition ability of APPS on DNA damage-induced p53 upregulation. When NIH3T3 cells were cultured with camptothecin (CPT), a DNA damage-inducer, p53 protein was significantly upregulated in a dose-dependent manner (Figure 4F). The APPS treatment, however, failed to inhibit p53 upregulation by CPT treatment, indicating that APPS is not able to suppress p53 expression in response to DNA damage. These results revealed that a VC derivative effectively normalized the cellular viability, O_2_^•−^ accumulation, and p53 upregulation via the direct anti-oxidant activity in *Sod1^−/−^* fibroblasts.

### 2.4. A Vitamin C Derivative Treatment Reversed Skin Atrophy Induced by Sod1 Loss in Vivo

**Figure 5:**
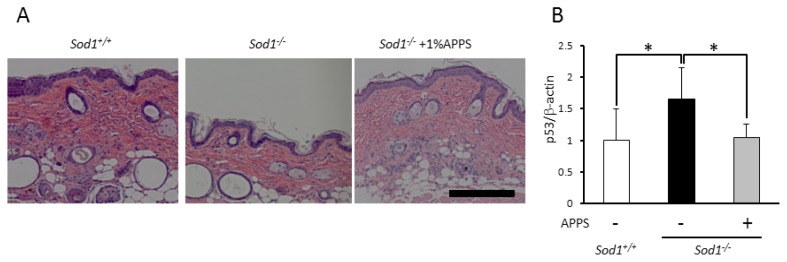
A VC derivative rescues skin phenotypes in *Sod1^−/−^* mice. (**A**) The hematoxylin and eosin staining of the back skin of *Sod1^−/−^* and *Sod1^+/+^* mice. *Sod1^−/−^* mice (5 months old) were transdermally treated with 1% APPS for 4 weeks. The scale bar represents 100 μm; (**B**) p53 mRNA expression was analyzed by quantitative PCR in skin tissues with or without APPS treatment (*n* = 6–7). These data indicate the mean ± SD; * *p* < 0.05.

We have previously reported that transdermal treatment with APPS reverses skin thinning in *Sod1^−/−^* mice [8,19]. Treatment with APPS also significantly reduced lipid peroxidation in the skin of *Sod1^−/−^* mice [19]. In the present study, we confirmed the beneficial effects of transdermal APPS treatment on skin atrophy in *Sod1^−/−^* mice (Figure 5A). In order to elucidate the rescue mechanism of APPS on skin pathology in *Sod1^−/−^* mice, we measured p53 expression using quantitative PCR. As shown in Figure 5B, treatment with APPS remarkably reduced p53 expression in the skin of *Sod1^−/−^* mice compared to non-treated *Sod1^−/−^* mice, suggesting that the anti-oxidant activity of APPS normalized skin pathologies by suppressing O_2_^•−^-mediated p53 activation *in vivo*.

### 2.5. Discussion

In the present study, we demonstrated that *Sod1^−/−^* fibroblasts showed decreased proliferative ability and increased apoptosis (Figure 1). Furthermore, *Sod1* loss induced O_2_^•−^ accumulation in both the cytoplasm and the mitochondria (Figure 2). Okado-Matsumoto reported that *Sod1* is distributed in the cytoplasm and in the intermembrane space of mitochondria [5] and Muller *et al.* found that complex III released O_2_^•−^ to both sides of the inner membrane in mitochondria isolated from skeletal muscles [20]. Recently, Jang *et al.* also reported increased ROS generated from isolated mitochondria in the skeletal muscle of *Sod1^−/−^* mice [21], suggesting that *Sod1* physiologically regulates not only cytoplasmic O_2_^•−^ generation, but also mitochondrial O_2_^•−^ release. In our preliminary experiments, we demonstrated that *Sod2^−/−^* fibroblasts showed an increased O_2_^•−^ accumulation in the mitochondria and in the cytoplasm, resulting in decreased proliferation. Interestingly, *Sod2* deficiency did not induce apoptosis in spite of increased O_2_^•−^ accumulation in the fibroblasts. Taken together, these results suggested that *Sod1* might protect from the apoptosis induced by intracellular O_2_^•−^ accumulation in fibroblasts.

Since oxidative DNA damage induced by such irritants as irradiation and chemicals promotes p53 upregulation and phosphorylation via a canonical ATM-p53 cascade [2], we investigated DDR such as phosphorylated H2AX (γH2AX) and p53 activation. As expected, *Sod1^−/−^* fibroblasts showed increased γH2AX with upregulation and phosphorylation of p53 at Ser^18^ (Figure 3C), whose residue is phosphorylated by ATM [2]. Moreover, p21, a p53 target gene, was significantly upregulated in *Sod1-*deficient fibroblasts (Figure 3C), thus suggesting that intracellular O_2_^•−^ accumulation stimulated the canonical DDR cascade including p53 activation leading to growth arrest and apoptosis in *Sod1^−/−^* fibroblasts. Accumulating evidence demonstrates that cellular p53 levels are primarily regulated by ubiquitin-mediated proteasomal degradation [22]. ATM phosphorylates both p53 at Ser^15^ (mouse Ser^18^) and MDM2, an endogenous E3-ligase for p53, at Ser^395^, resulting in an impairment of MDM2-mediated p53 degradation [22]. Since ROS-induced DDR promotes ATM activity, ATM-mediated MDM2 phosphorylation may stabilize p53 levels in *Sod1^−/−^* cells (Figure 3C). In contrast, Gajjar *et al.* recently reported that p53 mRNA-MDM2 interaction controls MDM2 nuclear trafficking and p53 activation following DNA damage. They demonstrated that ATM-dependent phosphorylation of MDM2 at Ser^395^ resulted in its recruitment to p53 mRNA, thereby stimulating p53 upregulation [23,24]. Thus, DDR-mediated p53 mRNA-MDM2 interaction may increase p53 mRNA in *Sod1^−/−^* skin tissues (Figure 5B). Further analyses are therefore needed to clarify the molecular mechanism of the upregulation of p53 in *Sod1*-deficient mice.

Interestingly, *Sod1^−/−^* fibroblasts also showed mitochondrial O_2_^•−^ accumulation and low ΔΨm (Figure 3A,B). Since excess ROS impairs ΔΨm [25], the cytoplasmic O_2_^•−^ accumulation caused by *Sod1* deficiency may induce the loss of ΔΨm resulting in the O_2_^•−^ accumulation in the mitochondria. Alternatively, since mitochondrial ROS also directly impairs ΔΨm [3], concomitant O_2_^•−^ accumulation in the mitochondria may cause decreased ΔΨm. In any case, O_2_^•−^-mediated ΔΨm decline may cause apoptosis. Recently, Jiang *et al.* reported that p53 repressed the expression of TCA cycle-associated malic enzymes ME1 and ME2 in cells [26]. Since both malic enzymes are important for NADPH production, lipogenesis, and glutamate metabolism, the upregulation of p53 may also directly impair mitochondrial function via ME1 and ME2 repression leading to ΔΨm decline.

In a mouse model, p53 activation (p53m allele) by genetic engineering induces accelerated aging including skin atrophy [27]. Gannon *et al.* reported that *Mdm2* loss in keratinocytes induced p53 upregulation resulting in epidermal stem cell senescence and premature aging phenotypes in mice [28]. In skin tissue, p53 also plays a crucial role in cellular fate and homeostasis. In the present study, we demonstrated that *Sod1* loss induced p53 upregulation in the fibroblasts and the skin due to intracellular O_2_^•−^ accumulation. We also demonstrated that antioxidants effectively rescued O_2_^•−^-p53 mediated cellular and skin pathologies *in vitro* and *in vivo*. These data indicate the possibility that antioxidant treatment is a promising strategy to delay skin aging by remarkably reducing the p53 upregulation in the skin.

It is widely known that VC is a soluble vitamin and an essential cofactor for post-translational modifications in collagen formation [29]. We previously reported that the oral administration of VC significantly improved low-turnover osteoporosis [9] as well as reduced behavioral deficits in the AD model mice [30]. However, the oral administration of VC failed to improve skin atrophies of *Sod1^−/−^* mice (unpublished results), indicating the low bioavailability and stability of VC in the skin tissues. Since VC exhibits both low stability and liposolubility, it is difficult to transdermally absorb into the skin. Actually, we showed that a transdermal treatment of VC itself failed to improve the skin atrophy of *Sod1^−/−^* mice [19]. To increase the stability and liposolubility of VC, various VC derivatives were developed for dermatologic application. One of the VC derivatives, APPS, is conjugated to a phosphate group and a long hydrophobic chain to enhance its stability and liposolubility. Transdermal treatment with APPS effectively normalized skin thickness in *Sod1^−/−^* mice through the suppression of oxidative damage and p53 upregulation (Figure 5). Treatment with APPS in wild-type mice showed no adverse effects in the skin tissues. Therefore, VC derivatives, including APPS, are powerful anti-aging agents for skin through an antioxidant effect.

## 3. Experimental Section

### 3.1. Animals

*Sod1^−/−^* mice were purchased from the Jackson Laboratory (Bar Harbor, ME, USA). All of the genotypes of Sod1 mice were assessed by PCR using genomic DNA isolated from the tail tip as described previously [9]. The animals were housed under a 12 h light/dark cycle and were fed ad libitum. The experimental procedures were approved by the Animal Care and Use Committee of Chiba University.

### 3.2. Cell Culture

The skin tissue specimens were dissected from 5-day-old *Sod1^−/−^* neonates. The primary dermal fibroblasts were isolated by dissociation in 0.2% collagenase type 2 (Worthington Biochemical Corporation, Lakewood, NJ, USA) at 37 °C for 60 min. The cells were cultured in α-MEM (Life Technologies Corporation, Carlsbad, CA, USA) supplemented with 20% fetal bovine serum (FBS), 100 unit/mL penicillin, and 0.1 mg/mL streptomycin at 37 °C in a humidified incubator with 5% CO_2_ and 1% O_2_. During experiments, the cells were cultured under a 20% O_2_ condition. The cells were treated with 10 μM L-ascorbyl 2-phosphate 6-palmitate (APPS, Showa Denko K. K., Tokyo, Japan) at the indicated times. Cell viability was measured by cell proliferation ELISA, BrdU (Roche Diagnostics K.K., Tokyo, Japan) or Cell Counting Kit-8 (Dojindo Laboratories, Kumamoto, Japan) according to the manufacturer’s instructions. NIH3T3 cells (RCB1862) were cultured in DMEM (Sigma-Aldrich inc., St. Louis, MO, USA) containing 10% FBS, 100 unit/mL penicillin, and 0.1 mg/mL streptomycin. The cells were treated with 20 μM APPS for 24 h. To induce DNA damage, NIH3T3 cells were treated with camptothecin (Enzo Life Sciences Inc., Tokyo, Japan) for 4 h.

### 3.3. Western Blotting

The fibroblasts were lysed in an NP-40 lysis buffer [50 mM Tris-HCl, pH 8.0; 150 mM NaCl; 1% NP-40; protease inhibitor (1 mM phenylmethanesulfonylfluoride and 2 μg/mL aprotinin); and phosphatase inhibitors (1 mM sodium fluoride and 1 mM β-glycerophosphate 2Na)]. The samples were placed at 4 °C for 20 min, vortexed, and centrifuged at 15,000 rpm for 10 min. The supernatants were collected, and 20 μg of each sample were loaded onto a 10% SDS-polyacrylamide gel. Antibodies against SOD1 (1:500, ADI-SOD-100-F, Enzo Life Sciences), SOD2 (1:500, ADI-SOD-200-F, Enzo Life Sciences), caspase3 (1:500, #9665, Cell Signaling Technology, Inc. MA, USA), cleaved caspase3 (1:500, #9664S, Cell Signaling Technology), p53 (1:200, #2524S, Cell Signaling Technology), human phospho-p53 at Ser^15^ (mouse Ser^18^) (1:200, #9284S, Cell Signaling Technology), and actin (1:1000, A2066, Sigma, St. Louis, MO, USA), and lamin A/C (1:500, #2035, Cell Signaling Technology) were used.

### 3.4. Flow Cytometry

The accumulation of intracellular O_2_^•−^ was detected using dihydroethidium (DHE, Life Technologies Corporation, Gaithersburg, MD, USA) and MitoSOX (Life Technologies Corporation), which are specific detectors of the O_2_^•−^ concentration in the cytoplasm and mitochondria, respectively [31]. To measure ΔΨm, the cells were stained with JC-1 dye, a ΔΨm probe (Life Technologies Corporation). The cells were incubated with 10 μM DHE, 5 μM MitoSOX, or 10 μM JC-1 for 30 min at 37 °C. Following incubation, the cells were trypsinized and resuspended in PBS. Apoptosis was measured using an FITC Annexin V Apoptosis Detection Kit I (BD Biosciences, San Jose, CA, USA) according to the manufacturer’s instructions. The fluorescence intensities were assessed using a flow cytometer (BD FACSCanto™ II, BD Biosciences, San Jose, CA, USA).

### 3.5. Histology

The fibroblasts were pre-incubated for 24 h with 1 mM *N*-acetylcysteine (NAC). The accumulation of intracellular O_2_^•−^ was detected using DHE. The cells were incubated with 10 μM DHE for 30 min at 37 °C. Following incubation, the cells were washed three times with buffer and then photographed using a Leica DFC300 FX camera (Leica Microsystems, Wetzlar, Germany) and the software application, Leica IM50 v4.0.

For the histological morphology analysis, the skin specimens from back tissues of *Sod1^−/−^* mice (5 months old) were dissected, fixed overnight in a 20% formalin neutral buffer solution (Wako Pure Chemical Industries, Ltd, Osaka, Japan), embedded in paraffin, and sectioned on a microtome at 4 μm thick by standard techniques. The hematoxylin and eosin staining was performed as described previously [8]. The thickness of the skin tissue was measured using Leica QWin V3 image software (Leica Microsystems).

### 3.6. Quantitative PCR

The total RNAs from the back skin were extracted with Trizol reagent (Life Technologies Corporation) according to the manufacturer’s instructions. The cDNA was synthesized from 1 μg of total RNA by reverse transcriptase (ReverTra Ace qPCR RT Master MIX, TOYOBO Osaka, Japan). Real-time PCR was performed on a Mini Opticon™ (Bio-Rad, Hercules, CA, USA) with SsoAdvanced SYBR Green Supermix (Bio-Rad, Redmond, WA, USA) according to the manufacturer’s instructions. The following primers were used for the analysis: β-actin, forward, 5′-GCCCTAGGCACCAGGGTGTGA-3′, and reverse, 5′-TCCTCAGGGGCCACACGCA-3′; Rn18s, forward, 5′-GTAACCCGTTGAACCCCATT-3′, and reverse, 5′-CCATCCAATCGGTAGTAGCG-3′; p53, forward, 5′-ACGCTTCTCCGAAGACTGG-3′, and reverse, 5′-AGGGAGCTCGAGGCTGATA-3′.

### 3.7. Statics

The statistical evaluations were performed using the two-tailed Student’s *t*-test for unpaired values. Any differences between the data were considered to be significant when the *p* values were less than 0.05. The data are represented as the means plus or minus the standard deviation.

## 4. Conclusions

Our results revealed that *Sod1* deficiency induced intracellular O_2_^•−^ accumulation in the cytoplasm and mitochondria resulting in decreased proliferation and increased apoptotic cell death in fibroblasts. Our findings also demonstrated that *Sod1* loss triggered the DNA damage response including p53 activation and promoted the mitochondrial dysfunction associated with low mitochondrial membrane potential. These results suggested that both mitochondrial dysfunction and p53 activation can cause the impairment of cell proliferation and the induction of apoptosis in *Sod1^−/−^* fibroblasts. Furthermore, antioxidant treatment effectively suppressed the p53 activation and improved the cellular and skin phenotypes caused by the *Sod1* deficiency *in vitro* and *in vivo*.
